# An Improved DSA-Based Approach for Multi-AUV Cooperative Search

**DOI:** 10.1155/2018/2186574

**Published:** 2018-12-02

**Authors:** Jianjun Ni, Liu Yang, Pengfei Shi, Chengming Luo

**Affiliations:** ^1^College of IOT Engineering, Hohai University, Changzhou 213022, China; ^2^Jiangsu Universities and Colleges Key Laboratory of Special Robot Technology, Hohai University, Changzhou 213022, China; ^3^Jiangsu Provincial Collaborative Innovation Center of World Water Valley and Water Ecological Civilization, Nanjing 210098, China

## Abstract

Multi-AUV cooperative target search problem in unknown 3D underwater environment is not only a research hot spot but also a challenging task. To complete this task, each autonomous underwater vehicle (AUV) needs to move quickly without collision and cooperate with other AUVs to find the target. In this paper, an improved dolphin swarm algorithm- (DSA-) based approach is proposed, and the search problem is divided into three stages, namely, random cruise, dynamic alliance, and team search. In the proposed approach, the Levy flight method is used to provide a random walk for AUV to detect the target information in the random cruise stage. Then the self-organizing map (SOM) neural network is used to build dynamic alliances in real time. Finally, an improved DSA algorithm is presented to realize the team search. Furthermore, some simulations are conducted, and the results show that the proposed approach is capable of guiding multi-AUVs to achieve the target search task in unknown 3D underwater environment efficiently.

## 1. Introduction

Autonomous underwater vehicle (AUV) has a wide range of applications in the field of science, commerce, and military, such as searching missing airplanes and ships wreckage, maritime rescuing, and exploitation of marine resources [1–4]. Multi-AUV system attracts more and more attentions recently, due to its high parallelism, robustness, and collaboration of high efficiency [5, 6]. Multi-AUV system can complete difficult tasks more rapidly and efficiently than a single AUV, so it is a very important development direction in the research field of AUV [7–9].

Target search is a very important and basic mission in the applications of multi-AUVs. Also, it is a very challenging task in the field of multiple AUVs. Lots of work has been done to deal with the target search problem. For example, Xiao et al. [10] presented a cooperative multiagent search algorithm to solve the problem of searching for a target on a 2D plane under multiple constraints. Li and Duan [11] proposed a game theoretic formulation for multiple unmanned aerial vehicle cooperative search and surveillance. Cai and Yang [12] proposed a novel potential field-based particle swarm optimization approach for a team of mobile robots to cooperatively search targets in unknown environments. Most of the existed methods are focused on the search problem for the ground mobile robot on 2D environments. However, there are obvious differences between the search tasks for the ground mobile robot and the underwater robot. The underwater environment is three-dimensional (3D) and has lots of uncertainties [13–15], which is more complicated than 2D ground environment. So, the general cooperative search approaches for the ground mobile robot cannot be used directly for multi-AUVs.

Much research has been done to deal with various tasks in the cooperative search task of multi-AUVs. For example, Zhu et al. [16] proposed a biologically inspired self-organizing map method for the dynamic task assignment and path planning of multi-AUV system. Cao et al. [17] proposed an integrated algorithm for a cooperative team of Multi-AUVs by combining the Glasius bioinspired neural network and bioinspired cascaded tracking control approach to improve search efficiency and reduce tracking errors. Yi et al. [18] studied the task assignment problem of a swarm of robots in 3D dynamic environments, and an improved approach that integrates the advantages and characteristics of biological neural systems is proposed. Li et al. [19] presented a bioinspired geomagnetic navigation method for AUV without using any a priori geomagnetic information. Those methods discussed above are all the key technologies of the multi-AUV search task, and the research results provided a good basic for the cooperative search task of multi-AUVs. However, few of those methods above considered the cooperative search task as a whole, and the search task for the dynamic targets is often ignored.

To complete the cooperative search task efficiently for multi-AUVs, the complexity of the search task in the 3D unknown underwater environment with great uncertainties should be considered. The safety of the AUVs and the cooperative efficiency are two main issues in the search task [20, 21]. Recently, many researchers have done lots of work in this field. For example, Ni et al. [22] proposed a partition and column parallel search strategy and a formation search control algorithm based on an improved spinal neural system in 3D underwater environment with obstacles. Abreu et al. [23] presented a coverage path planning technique for search operations, which takes into account the uncertainties of the vehicle position and detection performance. Cao et al. [24] studied the problem of the target search in 3D underwater environments, and an integrated strategy is proposed including the map building based on the Dempster-Shafer theory of evidence and the path planning based on a bioinspired neurodynamics model. However, there are still some shortcomings in the existing methods that should be solved, such as the low efficiency of the dynamic targets search in the complex 3D underwater environment.

The methods of cooperative search for multiple AUVs can be divided into two types according to the target information. One is based on known information of target prior distribution, such as heuristic search methods [25, 26]. The other is based on sensor information without any target information, such as the region search methods [27]. This paper is focused on the sensor information-based cooperative search task based on the swarm intelligence algorithm.

In the proposed approach, a novel integrated search method is proposed, including the random cruise strategy, the dynamic alliance construction, and the method to find the position of target. Firstly, a Levy flight-based random cruise strategy is proposed to give AUV a random walk to cruise in the underwater environment [28, 29]. In this study, the Levy flight algorithm is improved by an adjustment module based on fuzzy rules, to make it suitable for the movement characteristics of AUV and the complexity of the underwater environment [30]. After retrieving the target information, a dynamic alliance for the team search is determined using the SOM neural network algorithm [31, 32]. Then, an improved dolphin swarm algorithm- (DSA-) based approach is used to plan the path for each AUV to search the target [33, 34]. The cooperative search approach in this paper takes full consideration of the obstacles, making the search task more in line with the actual situation and improving the practicality of the method.

The main contributions of this paper are summarized as follows. (1) A cooperative search task in 3D unknown underwater environment is presented, which is completed by a multi-AUVs system. (2) An improved DSA-based method is proposed for multi-AUV target search, which is an integration of several methods, such as the fuzzy rules-based Levy flight algorithm for AUVs cruising and the improved DSA-based team search method. (3) The ability of the DSA-based method is improved. The calculation time of the algorithm is reduced, and the search efficiency and the adaptivity of the algorithm for AUVs in 3D underwater environment are increased. (4) Some simulations are conducted in 3D underwater environments, where the situations of the search task for static targets and dynamic targets are simulated. And the proposed algorithm is compared with the general dolphin swarm algorithm and PSO algorithm in these simulations.

This paper is organized as follows.  Section 2 presents the problem statement. The proposed cooperative search approach for multiple AUVs based on the improved DSA is given in Section 3.  Section 4 gives out the simulation studies and the result analysis. The performance of the proposed approach is discussed in Section 5. Finally, conclusions are given in Section 6.

## 2. Problem Statement

In this paper, the multi-AUV cooperative target search problem in unknown 3D underwater environment is studied. The technical details are not focused in this study, including the shape and movement of the AUV, environment detection, and communication problems. The search task in this paper is that an AUV system is used to find some targets in this underwater environment. The problems are introduced as follows:

1. The AUVs are labelled as *a*_*i*_, *i*=1,2,…*N*, and the set of targets are denoted as *t*_*i*_, *i*=1,2,…*M*. *N* is the number of the AUVs used in the search task, and *M* is the number of the targets.
2. The target has some information that can be detected by the AUVs (such as the infrared radiation of the heat source, the radiation of the radioactive source, or the odour of the odorous source), and the intensity of the target information in the environment is defined as follows:

(1) $$\begin{array}{c} I_{\text{t}}\left(t_{i},p_{\text{e}}\right)=\left\{\begin{array}{cc} S_{\text{t}}, & \text{if}\;\;D\left(p_{{\text{t}}_{i}},p_{\text{e}}\right)\leq 1,\\ \frac{S_{\text{t}}}{D\left(p_{{\text{t}}_{i}},p_{\text{e}}\right)}, & \text{if}\;\;1<D\left(p_{{\text{t}}_{i}},p_{\text{e}}\right)\leq R_{\text{t}},\\ 0, & \text{if}\;\;D\left(p_{{\text{t}}_{i}},p_{\text{e}}\right)>R_{\text{t}}, \end{array}\right. \end{array}$$

where *I*_t_ represents the intensity of target information; *p*_t_ and *p*_e_ are the coordinates of the target *T*_*i*_ and a point *P*_*i*_ in the environment, respectively; *S*_t_ is the largest information intensity; *R*_t_ represents the propagation radius of the target information; and function *D*(*p*_*i*_, *p*_*j*_) defines the distance between the position *p*_*i*_ and *p*_*j*_.

(3) Each AUV is considered as an omnidirectional robot, having a 360° visual capability and the abilities to communicate with other AUVs, recognize each other, identify the information of target, detect obstacles, and determine their locations in real time. For simplification without losing generality, the AUV can change the moving direction without delay. The movement speed of the AUV can be changed in a certain range around the standard speed (defined as *v*_a_).
(4) The AUVs have no knowledge about the environment and the locations of targets, except the number of targets to be searched. The closer the AUV is to the target, the bigger the intensity of the target information is. If the target is in the visual range of the AUV (denoted as *R*_v_, which is very small, because the underwater environment is dark), it is found and locked by this AUV.

**Figure 1:**
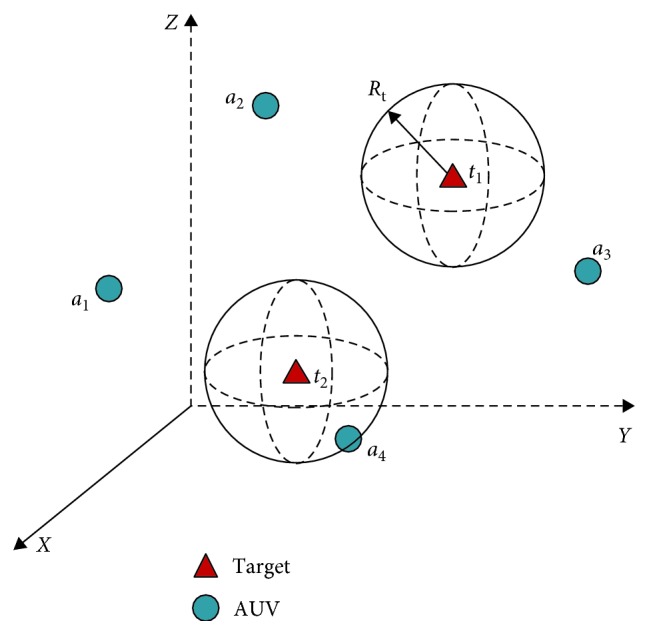
A simple example of underwater target search task based on multi-AUVs.

**Figure 2:**
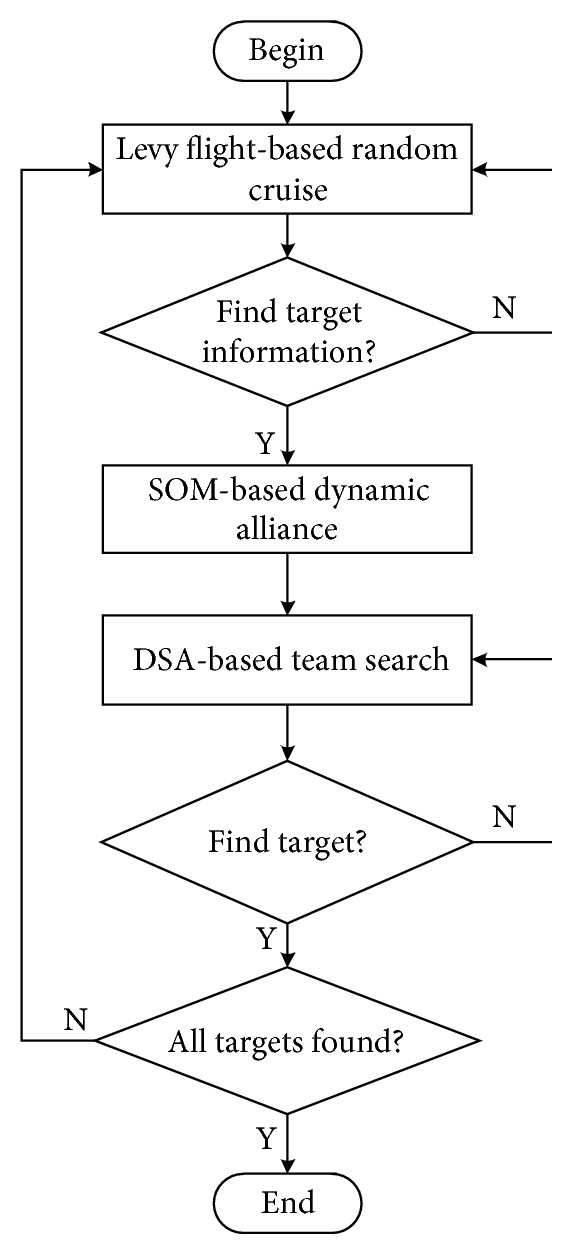
The work flow diagram of the search task in this study.

The search task of the targets based on multi-AUVs is shown in Figure 1. The work flow and the proposed solutions for the three main stages in this study are shown in Figure 2, which will be introduced in detail as follows.

## 3. Proposed Approach

In order to complete the cooperative search task for multi-AUV system in an unknown 3D underwater environment, some key problems should be solved efficiently, including the random cruise strategy, the dynamic alliance construction, and the way to find the position of target according to the target information. In this paper, a multi-AUV cooperative target search approach based on an improved DSA algorithm is proposed.

Before the introduction of the proposed approach, it is necessary to define some flags. One flag is denoted by *f*_1_(*a*_*i*_), indicating the AUV status as cruising, searching, or locking. Another flag is denoted by *f*_2_(*t*_*i*_), indicating the target status as unknown, known, or locked:

(2) $$\begin{array}{c} f_{1}\left(a_{i}\right)=\left\{\begin{array}{cc} 1, & \text{if}\;\;\mathrm{it}\;\;\mathrm{is}\;\;\mathrm{cruising,}\\ 2, & \text{if}\;\;\mathrm{it}\;\;\mathrm{is}\;\;\mathrm{searching,}\\ 3, & \text{if}\;\;\mathrm{it}\;\;\mathrm{is}\;\;\mathrm{locking}\;\;a\;\;\mathrm{target}, \end{array}\right.\\ f_{2}\left(t_{i}\right)=\left\{\begin{array}{cc} 1, & \text{if}\;\;\mathrm{it}\;\;\mathrm{is}\;\;\mathrm{unknown,}\\ 2, & \text{if}\;\;\mathrm{it}\;\;\mathrm{is}\;\;\mathrm{known,}\\ 3, & \text{if}\;\;\mathrm{it}\;\;\mathrm{is}\;\;\mathrm{locked}. \end{array}\right. \end{array}$$

### 3.1. Levy Flight-Based Random Cruise

When the AUV *a*_*i*_ does not find any target information, set *f*_1_(*a*_*i*_)=1. The AUV *a*_*i*_ is cruising randomly in the underwater environment, in which an appropriate random cruise strategy is crucial. In this paper, Levy flight is used to randomly change the position of the AUV. The Levy flight essentially provides a random walk for robots, while the random step length *L* is drawn from a levy distribution [35]:

(3) $$\begin{array}{c} \text{Levy}\left(L\right)∼L^{-\lambda },\;\left(1<\lambda \leq 3\right), \end{array}$$

where *λ* is an index. Then the next position of the AUV *a*_*i*_ can be decided by

(4) $$\begin{array}{c} p_{a_{i}}\left(t+1\right)=p_{a_{i}}\left(t\right)+\alpha \;⊕\;L, \end{array}$$

where *p*_*a*_*i*__(*t*) is the position of the AUV *a*_*i*_ at the time *t*; *α* > 0 is an adjustment parameter which should be related to the velocity of the AUV; ⊕ means element-by-element multiplication; *L* can be calculated by Mantegna's algorithm as [36]:

(5) $$\begin{array}{c} L=\frac{\mu }{{\left|\nu \right|}^{1/\beta }}, \end{array}$$

where *μ* and *v* are drawn from normal distributions.

**Figure 3:**
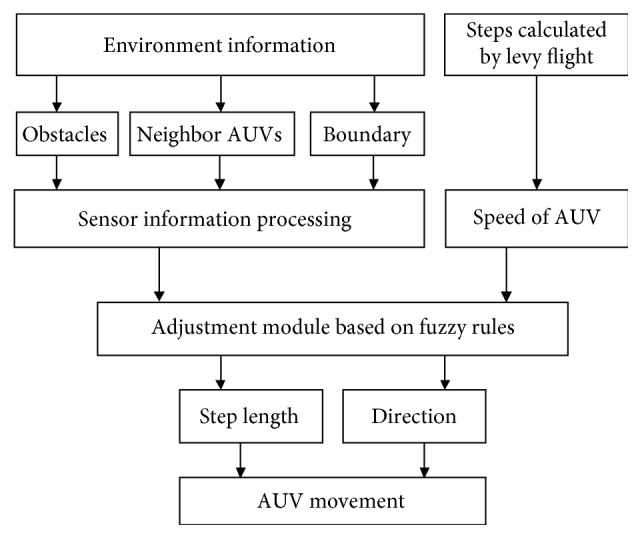
Flowchart of the adjustment module based on fuzzy rules for the movement of AUVs.

Based on the general Levy flight, the AUV can get a random path to search for target information. However, some important things are not considered by the general Levy flight algorithm, because it is often used in optimization algorithms. For example, the AUV may run out of the search area and collide with the obstacles and other AUVs, and the step length generated by the levy algorithm is not suitable for the movement of AUVs. To deal with these problems above, an adjustment module based on fuzzy rules is introduced into the general Levy algorithm. The structure of it is shown in Figure 3.

One of the inputs of the proposed adjustment module is the environment information (including the distance to the obstacles, the distance to the neighbor AUV, and the distance to the search area boundary, defined as OD, ND, and BD, respectively). To consider the real movement of the AUV, another input of the adjustment module is defined as follows:

(6) $$\begin{array}{c} \Delta L=L-v_{\text{a}}\;*\;\Delta t, \end{array}$$

where Δ*t* is the simulation step length. The outputs of the adjustment module are the adjustment for the step length *A*_l_ and the movement direction *A*_d_.

The adjustment module in this paper is based on some fuzzy rules. Considering the complexity of the underwater environment, the membership of the input variables OD, ND, and BD are divided as three fuzzy sets, which are {*N*, *M*, *F*} (representing near, middle, and far). The membership of variables Δ*L* and *A*_l_ are divided as five fuzzy sets, which are {NB, NM, *Z*, PM, PB}. The membership function used in this paper is Gaussian function. To make the AUV to find the target information efficiently, the *A*_d_ is defined as {ST, TU}, representing going straight and making a turn (the turning angle is random in the range of [−*π*, *π*]). Based on the experiences, total nine fuzzy rules are summarized for the adjustment module, and the style of these rules is as follows:

(7) $$\begin{array}{c} \text{If}\;\;\left(\text{OD}\;\;\mathrm{is}\;\;N\right)\;\;\mathrm{and}\;\;\left(\text{ND}\;\;\mathrm{is}\;\;N\right)\;\;\mathrm{and}\;\;\left(\text{BD}\;\;\mathrm{is}\;\;N\right)\;\;\mathrm{and}\;\;\left(\Delta \text{L}\;\;\mathrm{is}\;\;\mathrm{NB}\right),\\ \text{Then}\;\;\left({\text{A}}_{\text{l}}\;\;\mathrm{is}\;\;\mathrm{PB}\right)\;\;\mathrm{and}\;\;\left({\text{A}}_{\text{d}}\;\;\mathrm{is}\;\;\mathrm{TU}\right). \end{array}$$

In this stage, all AUVs move randomly in the search area to detect the target information until an AUV finds target information. Then it goes to the next stage.

### 3.2. Dynamic Alliance Based on SOM Neural Network

After the AUV *a*_*i*_ finds the target information, the flag of this AUV is set as *f*_1_(*a*_*i*_)=2 and the flag of the detected target *t*_*j*_ is labelled as *f*_2_(*t*_*j*_)=2. Before the detected target *t*_*j*_ is found, the AUV who finds the target information should construct a dynamic AUV alliance to search the target faster and more effectively, which can be seen as a task assignment problem.

**Figure 4:**
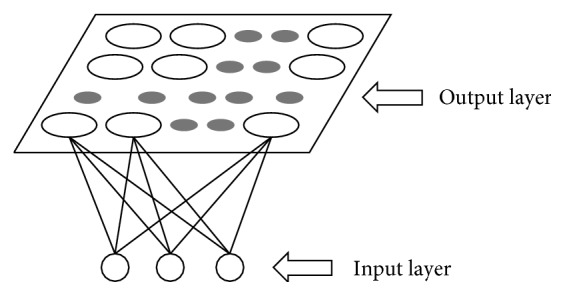
SOM neural network structure of the proposed approach for the dynamic alliance of AUVs.

In this study, a dynamic alliance assignment strategy based on SOM neural network is proposed. The SOM neural network contains two layers, namely, the input layer and the output layer (Figure 4). Each neuron in the output layer of the neural network gets the opportunity to respond to the input through competition. Finally, only one neuron becomes the winner. SOM neural network is competitive, cooperative, and self-organized, which can be used to solve the task assignment problem for multi-AUV system efficiently.

In the proposed SOM neural network, the input layer is made up of a neuron *A*_*i*_=(*x*_*a*_*i*__, *y*_*a*_*i*__, *z*_*a*_*i*__), which represents the coordinate of the AUV *a*_*i*_ in the 3D underwater environment. And the coordinates of the other AUVs are denoted as the output layer neurons: *A*_*k*_=(*x*_*a*_*k*__, *y*_*a*_*k*__, *z*_*a*_*k*__), *k*=1,…, *N*, *k* ≠ *i*. Every neuron of the output layer is fully connected to the neurons of the input layer, and for an input neuron, there is not only one winner.

Winners are chosen during the iterations, and the number of winners depends on the size of AUV team. For a given goal as an input, the output neurons compete to be the winner in an iteration according to a specified criterion described as [37]:

(8) $$\begin{array}{c} \left[A_{i},A_{k}\right]⇐\min\left\{D\left(A_{i},A_{k}\right),\;k=1,\ldots ,N,\;\;k\neq i,\;\;\mathrm{and}\;\;\left\{A_{k}\right\}\in \Omega \right\}, \end{array}$$

where [*A*_*i*_, *A*_*k*_] denotes that the *A*_*k*_th neuron from the *A*_*i*_th group of the output neurons is the winner; *Ω* is the set of neurons that have not been the winner yet in an iteration. If the *A*_*k*_th neuron is chosen to be a winner, the flag of the related AUV *a*_*k*_ is set as *f*_1_(*a*_*k*_)=2. All the winner AUVs with *f*_1_(*a*_*i*_)=2 construct the dynamic alliance.

### 3.3. Team Search Based on an Improved DSA

In this stage, AUVs in each team will search for the exact location of the target based on the intensity of the information, which is a cooperative search problem by optimal solution. Considering the complex underwater environment and the cooperation between the AUVs in a team, an improved dolphin swarm algorithm (DSA) is proposed for multi-AUV cooperative search, which is an efficient global search method to solve various optimization problems, by simulating the dolphin's actual predatory process [33]. The main reason to use DSA method in this paper is that the DSA has better global search ability, better stability, and higher convergence speed, compared with the conventional evolutionary algorithms. To make the DSA method more efficient for the team search, some improvements are presented in this paper, which are introduced as follows.

In the proposed DSA-based method, each dolphin represents an AUV, in the 3D search space of the underwater environment. The dolphin is defined as Dol_*i*_, (*i*=1,…, *N*), and its position is *p*_*i*_=(*x*_*i*_, *y*_*i*_, *z*_*i*_), which is the same as the coordinate of the AUV *a*_*i*_. For each dolphin Dol_*i*_, there are two corresponding variables *Q*_*i*_(*i*=1,…, *N*) and *K*_*j*_(*j*=1,…, *G*), where *Q*_*i*_ and *K*_*j*_ represent the optimized solution obtained by the *i*th dolphin Dol_*i*_ and by all the dolphins of the *j*th group in a single search time, respectively. *G* is the number of the dolphins in one group, namely the size of the AUV team. The fitness function Fitness(·) is the basis for judging whether the position is better, which is defined as follows:

(9) $$\begin{array}{c} \text{Fitness}\left(p\right)=\overset{M}{\underset{j=1}{\sum }}I_{\text{t}}\;\left(t_{j},p\right)-\omega \cdot \left[\text{ObstacleCheck}\left(p\right)+\text{NeighborCheck}\left(a_{i},p\right)\right], \end{array}$$

where *I*_t_(*t*_*j*_, *p*) is the information intensity of the target *t*_*j*_; *ω* is the parameter to adjust the effect on targets by obstacles or other AUVs; ObstacleCheck(*p*) is a function to check whether there is an obstacle, and NeighborCheck(*a*_*i*_, *p*) is a function to judge whether there are other AUVs too close to be collided. The two functions are defined as follows:

(10) $$\begin{array}{c} \text{ObstacleCheck}\left(p\right)=\left\{\begin{array}{cc} 1, & \text{if}\;\;\mathrm{there}\;\;\mathrm{is}\;\;\mathrm{an}\;\;\mathrm{obstacle}\;\;\mathrm{at}\;\;p,\\ 0, & \text{otherwise,} \end{array}\right.\\ \text{NeighborCheck}\left(a_{i},p\right)=\left\{\begin{array}{cc} 1, & \text{if}\;\;\exists D_{ik}<D_{\text{safe}},\;\;k=1,\ldots ,N,k\neq i,\\ 0, & \text{otherwise,} \end{array}\right. \end{array}$$

where *D*_safe_ is the safe distance of two AUVs to avoid colliding together.

There are four pivotal phases in DSA-based search process, namely, explore phase (namely, the search phase; to distinguish between the search phase of DSA and the search task of the multi-AUVs system, in this paper, we name it as explore phase), call phase, reception phase, and predation phase. The details of each phase are introduced as follows:

1. Explore phase. In this phase, each dolphin explores its nearby area by making sounds *S*_*i*_=[*S*_*i*_^*X*^, *S*_*i*_^*Y*^, *S*_*i*_^*Z*^], *i*=1,…, *H*, towards *H* random directions, where *S*_*i*_=speed, and speed is a constant representing the speed attribute of sound. Within the maximum explore time *T*_M_, the sound *S*_*j*_ that the dolphin Dol_*i*_ makes at the *t*th time will search for a new solution *X*_*ij*_^*t*^:

(11) $$\begin{array}{c} X_{ij}^{t}=p_{i}+S_{j}\times T_{\text{s}}, \end{array}$$

where *T*_s_=*T*_M_/*τ*, and *τ* is the total explore times for the dolphin. Then, the value of *Q*_*i*_ for the Dol_*i*_ at the *t*th time is calculated by

(12) $$\begin{array}{c} Q_{i}=X_{ih}^{t}⇐\text{Fitness}\left(X_{ih}^{t}\right)=\underset{j=1,\ldots ,H}{\max}\left\{\text{Fitness}\left(X_{ij}^{t}\right)\right\}, \end{array}$$

And, the value of *K*_*i*_ for Dol_*i*_ can be obtained:

(13) $$\begin{array}{c} K_{i}=\left\{Q_{\text{u}}\left|\forall Q_{\text{u}}\in \Pi_{i}\right.,\;\;\mathrm{Fitness}\left(Q_{\text{v}}\right)\leq \text{Fitness}\left(Q_{\text{u}}\right),\;\;Q_{\text{v}}\in \Pi_{i}\right\}, \end{array}$$

where Π_*i*_ means the set of the *Q* of the *i*th dolphin during the explore time *T*_M_.

**Figure 5:**
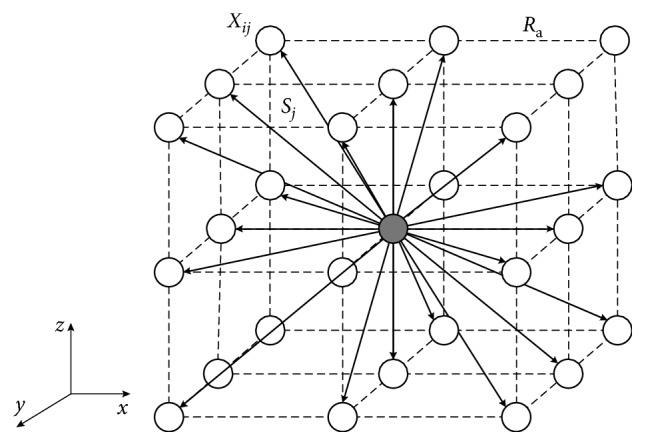
The 3D moving direction model of the DSA-based team search for multi-AUVs.

In the multi-AUV target search task, the AUV's motion characteristic in 3D underwater environment should be considered. In the general DSA-based method, the directions of sound are random, which will reduce the effects of the cooperative search. To deal with this problem, the random directions of sounds in the DSA-based method are replaced by a 3D dynamic moving directions model, which is shown in Figure 5.

In the proposed direction model, the core of it is the position of AUV, *R*_a_ is the detection range of the onboard sensors, and *S*_*j*_ represents the *j*th sound direction. Since all the possible directions are considered in the direction model, the maximum explore time *T*_M_ can be not considered and AUV only needs to search for the optimal direction once in a single explore time. Then, the new solution *X*_*ij*_ in equation (11) can be calculated by

(14) $$\begin{array}{c} X_{ij}=p_{i}+S_{j}\times R_{\text{a}}. \end{array}$$

(2) Call phase and reception phase. In the call phase, each dolphin will make sounds to inform other dolphins of its result in explore phase. In the reception phase, dolphins will determine if they can receive information from other dolphins, according to the transmission time matrix MT. In this study, for simplification, it is assumed that the communication between AUVs is normal; that means the sounds information from the call phase can be received by all dolphins in reception phase.

Then in these two phases, the *K*_*i*_ of the *i*th dolphin is updated as follows:

(15) $$\begin{array}{c} K_{i}=\left\{\begin{array}{cc} K_{j}, & \text{If}\;\;\mathrm{Fitness}\left(K_{i}\right)<\text{Fitness}\left(K_{j}\right),\\ K_{i}, & \text{otherwise,} \end{array}\right. \end{array}$$

where *K*_*j*_ is the valued of *K* obtained from all the dolphins in the same group.

(3) Predation phase. In this phase, the dolphins need to update their position according to *K*_*i*_ calculated by the previous phases. However, the *K*_*i*_ in one group is same based on the general DSA method, which may cause the collision between the AUVs in the same team. To deal with this problem, combined with the AUV's motion characteristics and the underwater environment, an adaptive reference point (RP) is proposed in this paper as follows:
  (16) $$\begin{array}{c} \text{RP}=\left\{\begin{array}{cc} K_{i}+\eta \times D\left(p_{i},K_{i}\right)\times \left(1-\frac{2}{\varepsilon }\right), & \text{If}\;\;D\left(p_{i},K_{i}\right)<R_{\text{a}},\\ Q_{i}+\eta \times D\left(Q_{i},K_{i}\right)\times \left(1-\frac{2}{\varepsilon }\right), & \text{otherwise,} \end{array}\right. \end{array}$$
  where *ε* > 2 is a constant; and *η* is an arbitrary unit vector. Then, the updated formula of the proposed algorithm is replaced by
  (17) $$\begin{array}{c} p_{i}\left(\text{new}\right)=\left\{\begin{array}{cc} \text{RP,} & \text{If}\;\;D\left(p_{i},\text{RP}\right)<v_{\text{a}},\\ p_{i}+\frac{\left(\text{RP}-p_{i}\right)}{D\left(p_{i},\text{RP}\right)}\times v_{\text{a}}, & \text{otherwise,} \end{array}\right. \end{array}$$

After all the dolphins get their new position *p*_*i*_(new), then update *K*_*i*_ by

(18) $$\begin{array}{c} K_{i}=\left\{\begin{array}{cc} p_{i}\left(\text{new}\right), & \text{If}\;\;\mathrm{Fitness}\left(K_{i}\right)<\text{Fitness}\left(p_{i}\left(\text{new}\right)\right),\\ K_{i}, & \text{otherwise,} \end{array}\right. \end{array}$$

**Figure 6:**
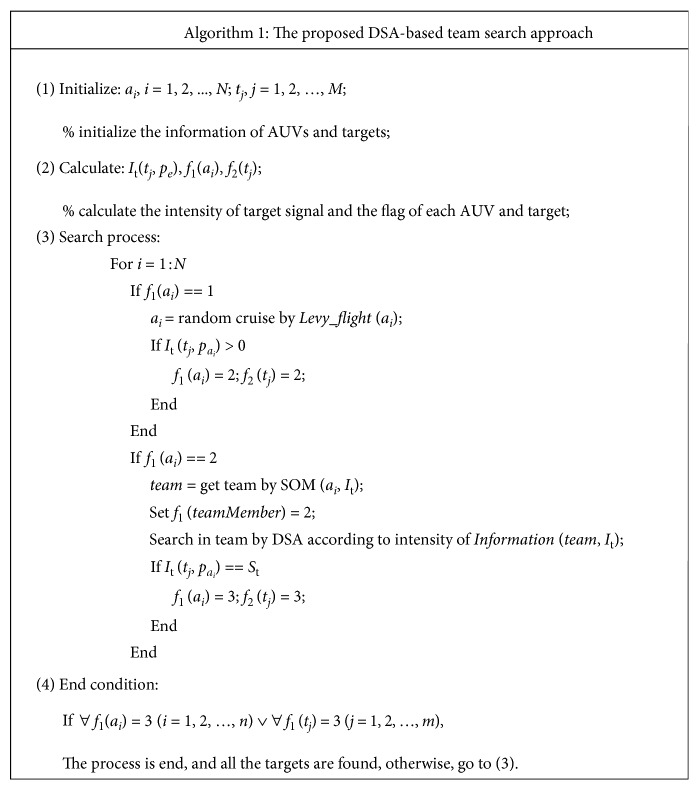
The pseudocode of the proposed DSA-based team search approach.

All the AUV will move towards the new position *p*_*i*_(new), until the target *t*_*j*_ is found. Otherwise, the DSA-based method will go to the explore phase again. The pseudocode of the whole proposed approach is shown in Figure 6 and its work flow is summarized as follows.

**Step 1 .**

The AUVs cruise in the unknown environment randomly using Levy flight algorithm, and detect the target information.

**Step 2 .**

If the target information is detected, a dynamic AUV alliance to search the target is constructed by the SOM neural network algorithm.

**Step 3 .**

Each AUV in the dynamic alliance is guided to find the position of the target using the dolphin swarm algorithm.

**Step 4 .**

Once a target is found by an AUV, then an AUV in the alliance will follow the tracks of this target, and others in the same alliance go to Step1.

**Step 5 .**

If all the targets are found, the search task is ended.

## 4. Simulation Experiments

To demonstrate the effectiveness of the proposed approach for cooperative target search of multi-AUVs in unknown 3D environment, some simulations are carried out by a computer with 4G RAM and i5-2450M 2.5 GHz CPU at the platform of MATLAB. To simplify the realization, the assumptions in this study are as follows: (1) The AUVs and targets are assumed as points without any shapes. (2) The obstacles are enlarged properly in the simulations to deal with the problem of the real shape and size of AUVs. (3) The targets are assumed to move randomly in the environment and the AUV velocity is greater than the target velocity; otherwise, it will be difficult to find the targets. In the simulations, the step length of AUV is 4 m and the step length of target is 2 m. (4) AUVs, obstacles, and target locations are randomly deployed in the 3D underwater environment.

The parameters in all the simulations are the same and given in Table 1. The size of the environment is 100*∗*100*∗*100 · (m^3^). To show the advantages of the proposed improved DSA-based algorithm (I-DSA), it is compared with the general PSO search algorithm (PSO) and the general DSA method (G-DSA). During the conduction of the target search task, the methods for the cruise stage and the dynamic alliance of the three approaches are same, expect the methods for the final team search are different. In the general PSO-based team search method, the main parameters are *c*_1_ and *c*_2_, which are the cognitive and social scaling factors [38] and are set as *c*_1_=1.2 and *c*_2_=1.2 in this study. The G-DSA-based method has the same parameters as the I-DSA-based method to have comparability, except that the G-DSA generates the directions randomly and has multiple iterations in the updating for *K*.

### 4.1. The Targets are Static

**Figure 7:**
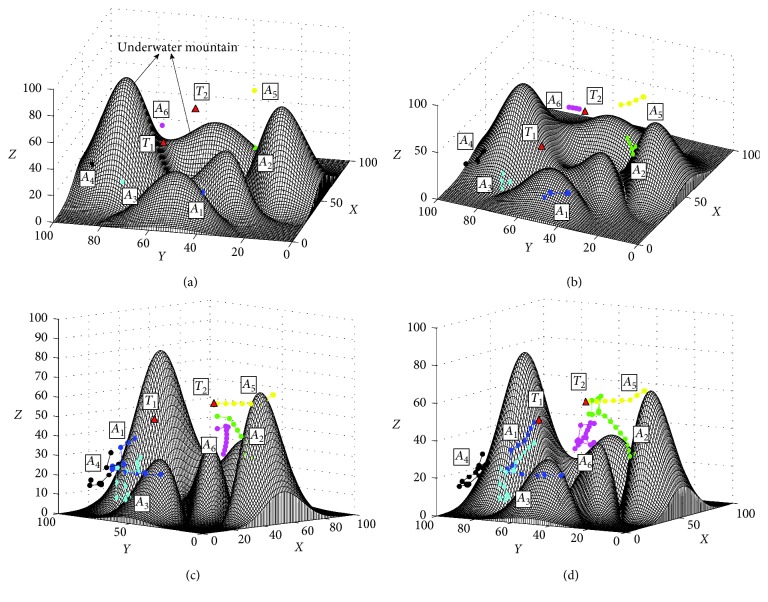
The search process of static targets based on the I-DSA method. (a) Initial positions of AUVs and the targets, view = (−77°, 32°). (b) At the 14th step, view = (−65°, 48°). (c) At the 33th step, view = (−44°, 8°). (d) Final trajectories, view = (−61°, 10°).

**Figure 8:**
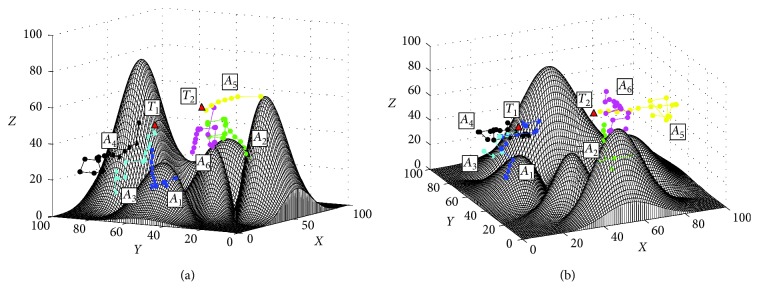
The final trajectories based on the PSO method and the G-DSA method. (a) Based on the G-DSA method, view = (−60°, 10°). (b) Based on the PSO method, view = (−25°, 32°).

In order to test the basic performance of the proposed approach, the first simulation of searching for the static targets is conducted. The search process based on the proposed I-DSA approach is shown in Figure 7.  Figure 7(a) shows the initial positions of AUVs and the targets. The initial positions of the AUVs are *A*_1_=(10,40,25), *A*_2_=(60,30,30), *A*_3_=(15,75,25), *A*_4_=(25,90,30), *A*_5_=(80,35,60), and *A*_6_=(85,75,25). And the initial positions of targets are *T*_1_=(25,60,50) and *T*_2_=(60,55,55).  Figure 7(d) shows the final trajectories based on the proposed method. The results of the comparison experiments are shown in Figure 8 and Table 2. In this study, an index is used to compare the comprehensive performance of the search methods, namely, the computation efficiency *C*_E_, which is defined as follows:

(19) $$\begin{array}{c} C_{\text{E}}=\frac{{\text{Total}}_{\text{time}}}{{\text{Total}}_{\text{steps}}}, \end{array}$$

where Total_time_ is the total time used to find all the targets and Total_steps_ is the total steps of all the AUVs used in the search task. The less the value of *C*_E_, the higher computation efficiency of the search method.

The results in Figure 7 show that the proposed method can find the targets effectively. At first time, the AUV knows nothing about the obstacles and targets in underwater environment, so each AUV cruises in the underwater environment randomly. Then, the information of *T*_2_ is detected by AUVs, and an alliance searching for *T*_2_ is formed by the AUVs *A*_2_, *A*_5_, and *A*_6_ (Figure 7(b)). When the target *T*_2_ is found and locked by *A*_5_ (Figure 7(c)), the other two AUVs in this alliance cruise randomly again to search the other targets. Finally, two targets are found and the search task is finished (Figure 7(d)). The results in Figure 8 and Table 2 show that all the three methods can find the static targets efficiently. Because the targets are static, the G-DSA method needs less steps and the length of it is less than the proposed I-DSA method. However, the computation efficiency of the G-DSA is less than the proposed method.

### 4.2. The Targets are Dynamic

**Figure 9:**
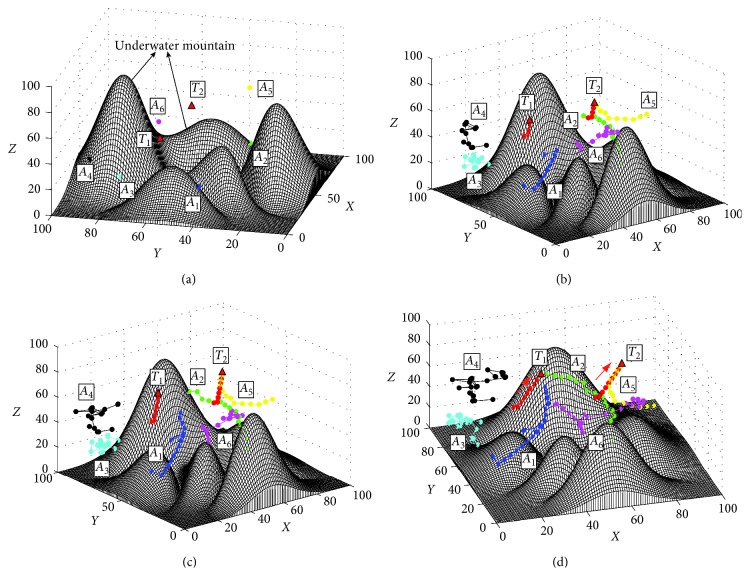
The search process of dynamic targets based on the I-DSA method. (a) Initial positions of AUVs and the targets, view = (−77°, 32°). (b) At the 27th step, view = (−37°, 28°). (c) At the 41th step, view = (−38°, 30°). (d) Final trajectories, view = (−17°, 44°).

**Figure 10:**
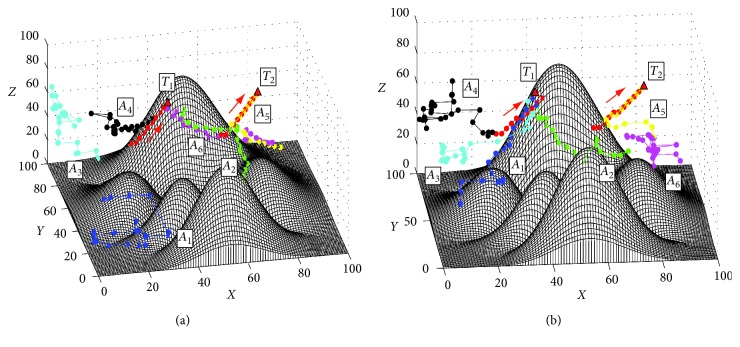
The final trajectories based on the PSO method and the G-DSA method. (a) Based on the G-DSA method, view = (−12°, 44°). (b) Based on the PSO method, view = (−6°, 32°).

To further test the performance of the proposed approach in the dynamic targets search task, this simulation is conducted. In this simulation, the targets can move randomly in the underwater environment. The results of the proposed approach are shown in Figure 9 and the initial positions of the AUVs and targets are the same as those of the static simulation (Figure 9(a)). The final trajectories based on the PSO method and the G-DSA method in this simulation are shown in Figure 10. The results of this simulation are listed in Table 3.

In the search process based on the proposed method, the target *T*_2_ is searched by the alliances *A*_2_, *A*_5_, and *A*_6_ firstly. Then, *T*_2_ is found and locked by *A*_5_. This process is similar with that of the static simulation (Figure 9(b)). However, the AUVs *A*_2_ and *A*_6_ will construct a new alliance with *A*_1_ to search *T*_1_, when the information of *T*_1_ is detected. Meanwhile, *A*_5_ moves following *T*_2_ (Figure 9(c)). Finally, *T*_1_ is found by *A*_2_, the search task finished, and the final positions of targets are *T*_1_=(41,74,64) and *T*_2_=(76,69,69) (Figure 9(d)). The results of this simulation show that the proposed approach can search the dynamic targets in unknown environment successfully with a relative smooth path than the G-DSA method and the PSO method (Figures 9(d) and 10). In addition, the data in Table 3 show that the proposed approach has greater superiority with the shortest path, the shortest time, and the highest computation efficiency in the dynamic target search task than the other two methods, which shows the proposed method has a higher efficiency in the dynamic target search task.

## 5. Discussions

The results of the simulations in Section 4 show that the proposed method can achieve the multi-AUV cooperative search task effectively in unknown 3D environment and shows great superiority comparing with the general DSA and PSO algorithm. Some performances of the proposed approach are discussed in this section.

**Figure 11:**
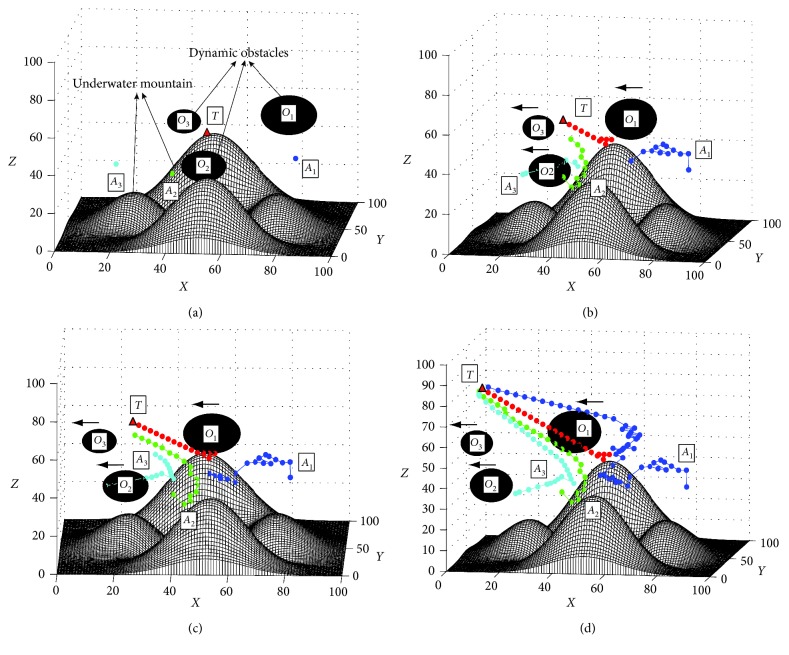
The search process of target based on the I-DSA method in a dynamic environment. (a) Initial positions of AUVs and the targets, view = (6°, 16°). (b) At the 32th step, view = (11°, 12°). (c) At the 44th step, view = (6°, 10°). (d) Final trajectories, view = (9°, 10°).

In order to test the effect of the proposed approach in searching target under a very complex situation, a simulation is conducted, where the parameters of the proposed approach are the same as those in Section 4, except there are some dynamic obstacles in the environment (the step length of the dynamic obstacle is 3 m). In this simulation, the number of AUVs and targets are 3 and 1, respectively, to show the target search process clearly. The initial positions of AUVs are *A*_1_=(80,75,30), *A*_2_=(40,25,35), and *A*_3_=(15,75,25), and the initial position of target is *T*=(50,52,49) (Figure 11(a)). The final path generated by the proposed approach is shown in Figure 11(d). In this simulation, the total time for the search task is 15.580 (s), and the path length of all AUVs is 465.19 m. The computational efficiency of the proposed approach in this task is 0.146, which increases obviously compared with the tasks in static environment. The main reason is that the proposed method should compute the environment in real-time during the cruise stage, which need much time in a dynamic environment. In spite of this, the results of this simulation show that the AUVs based on the proposed approach can find the target efficiently and avoid the moving obstacles simultaneously, in a dynamic unknown 3D environment (Figures 11(b)–11(d)).

**Figure 12:**
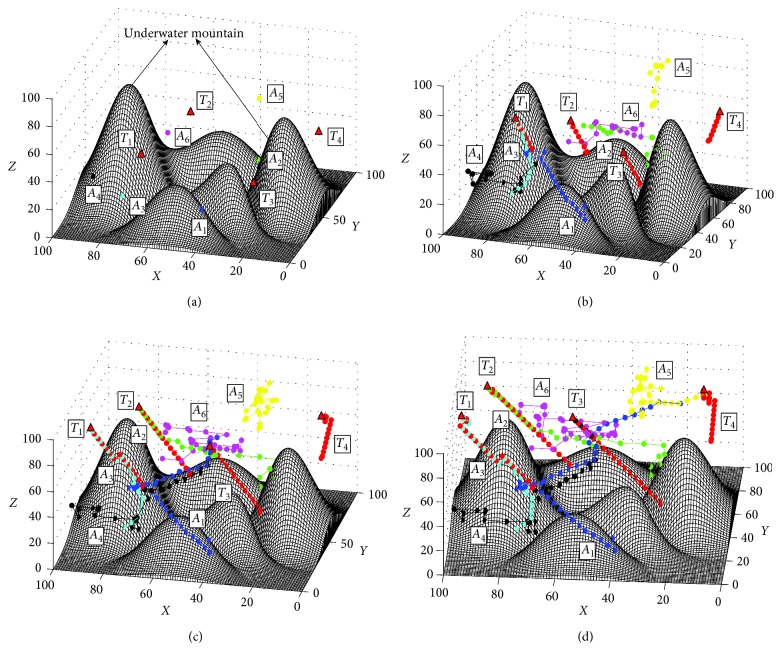
The search process of much more targets based on the I-DSA method. (a) Initial positions of AUVs and the targets, view = (−75°, 34°). (b) At the 32th step, view = (−70°, 28°). (c) At the 44th step, view = (−78°, 38°). (d) Final trajectories, view = (−86°, 44°).

To illustrate the extensive applications of the proposed algorithm, a simulation is carried out with much more targets. The environment and the parameters of the proposed approach are the same as those in Section 4, except the number of targets is increased to 4. The initial positions of the AUVs are the same with Section 4, and the initial positions of targets are *T*_1_=(25,70,50), *T*_2_=(65,60,30), *T*_3_=(35,25,30), and *T*_4_=(80,10,40) (Figure 12(a)). The results of the search process are shown in Figure 12. The total time of the search process is 31.326 (s), and the path length of all AUVs is 851.47 m. The computational efficiency of the proposed approach in this task is 0.181; the main reason is that much time is needed to find the target information, and the cooperative performance will decrease obviously when the ratio of the number of target with the number of AUVs decreases (Figures 12(b)–12(d)). The results of this simulation show that the proposed approach can deal with this challenging task. In addition, this simulation proves that multiple AUVs can increase the search efficiency, and the search efficiency and success rate will be increased greatly if there are much more AUVs joining the task.

## 6. Conclusion

The multi-AUV cooperative target search problem in unknown 3D underwater environment is studied in this paper, and a novel integrated method is proposed. In the proposed method, an improved Levy flight algorithm is used for the AUV random cruise in the unknown environment and the SOM neural network algorithm is used to construct the dynamic alliance for the AUVs which find the target information. And an improved dolphin swarm algorithm (DSA) is proposed to realize the final team search for targets. The proposed method can deal with the problems in the cooperative search task efficiently under various situations, such as the targets are dynamic and there are some moving obstacles in the environment. Furthermore, the movement characteristics of the AUV are considered in the proposed search method, which make it easy to apply the proposed method for real applications of target search task by multi-AUVs. In the future work, the real experiments for multiple AUVs cooperative search will be conducted to test the practical performance of the proposed method. In addition, some other bioinspired methods will be studied, to realize targets search by multi-AUVs more efficiently.

## Figures and Tables

**Table 1:** Parameters of the proposed method and the simulation experiments.

Parameters	Values	Remarks
*N*	6	Number of search AUVs
*M*	3	Number of targets
*R*_a_	10 m	Detection range of AUV
*R*_v_	1 m	Visual range of AUV
*R*_t_	20 m	Propagation radius of target information
*A*	1	Parameter of Levy flight in (4)
*β*	1.5	Parameter of Levy flight in (5)
*ω*	1000	Parameter of the fitness function in (9)
*ε*	2.2	Parameter of DSA method in (15)

**Table 2:** Parameters of the proposed method and the simulation experiments.

Search method	Length of path (m)	Total steps	Total time (s)	Computation efficiency
I-DSA	401.26	86	8.819	0.102
G-DSA	379.21	83	9.496	0.114
PSO	473.25	86	11.570	0.135

**Table 3:** Parameters of the proposed method and the simulation experiments.

Search method	Length of path (m)	Total steps	Total time (s)	Computation efficiency
I-DSA	544.80	110	12.506	0.113
G-DSA	617.12	130	18.287	0.141
PSO	703.14	128	19.143	0.150

## Data Availability

The data used to support the findings of this study are included within the article.
